# Comparison between PSMA PET/CT and MRI for Characterizing *Hepatocellular carcinoma*: A Real-World Study

**DOI:** 10.3390/tomography9010011

**Published:** 2023-01-13

**Authors:** Veronica Chi Ken Wong, Joshua Yip, Vincenzo Fragomeli, Martin Weltman, Han Loh, Ken Le, Diep Nguyen, Chuong Bui, Robert Mansberg

**Affiliations:** 1 Department of Nuclear Medicine and PET, Nepean Hospital, Kingswood, NSW 2747, Australia; 2 Faculty of Medicine and Health, The University of Sydney, Sydney, NSW 2006, Australia; 3 Department of Gastroenterology and Hepatology, Nepean Hospital, Kingswood, NSW 2747, Australia

**Keywords:** *Hepatocellular carcinoma*, PSMA PET/CT, liver

## Abstract

Prostate specific membrane antigen (PSMA) is expressed by hepatocellular carcinoma (HCC). PSMA PET/CT has potential as an imaging agent for the detection of HCC including early diagnosis and monitoring for recurrence following surgical resection. This study aims to compare PSMA PET to standard surveillance imaging in the detection of HCC. Patients with suspected or treated HCC were prospectively recruited from a tertiary hospital outpatient clinic. In addition to routine surveillance imaging as recommended by the multidisciplinary team, a PSMA PET/CT was performed. Imaging and clinical characteristics were compared over a follow-up period of up to 12 months. In a cohort of 19 patients with known HCC or suspected recurrent HCC, PSMA PET/CT had similar efficacy to MRI for the detection of HCC, with a sensitivity of 91% and a specificity of 70% and sensitivity of 87% and a specificity of 73% for PSMA PET/CT and MRI, respectively. PSMA PET/CT had a higher negative predictive value of 90%. In this relatively large single centre study, PSMA is shown to have promising equivalence in performance and its role should be further evaluated in multi-centre prospective trials.

## 1. Introduction

Worldwide, hepatocellular carcinoma (HCC) is the most common primary liver malignancy, the sixth most common neoplasm and the third leading cause of cancer death with an overall increasing incidence and a 5-year survival of 18% [1,2,3]. Earlier diagnosis of new or recurrent HCC in at-risk patients provides the best opportunity for effective treatment and improves long-term disease-free survival [1]. Currently, anatomic imaging relies on change in size, contrast enhancement and wash-out characteristics to diagnose lesions suspicious for HCC [4]. Oftentimes, treatment decisions, including the use of microwave ablation or targeted chemotherapy delivery, may be made based solely on imaging characteristics without a confirmatory histopathological diagnosis. Magnetic resonance imaging (MRI) with Gadoxetate disodium is considered the gold standard for imaging for HCC; however, access to this is limited due to excessive cost and limited availability of MRI. Surveillance programmes to detect new or recurrent HCC in at-risk individuals currently involve using ultrasound (US) and an alpha feto-protein (AFP) measurement and is supported by variable quality evidence of possible mortality benefit [5,6]. Anatomic imaging can also be limited by atypical imaging characteristics, reduced resolution in small lesions and is complicated by altered parenchymal architecture on a background of significant liver cirrhosis and patient factors including body habitus and previous treatment.

Prostate specific membrane antigen (PSMA) can be expressed in both benign and malignant liver conditions, including approximately 90% of HCC in in-vitro studies and small case series [7,8]. Therefore, additional assessment of a suspected tumour with a molecular probe such as with PSMA PET may help guide the diagnosis of HCC. This exploratory study aims to compare Ga-68 HBED-CC PSMA positron emission tomography/computed tomography (PET/CT) to conventional imaging with magnetic resonance imaging (MRI) and triple phase CT in patients with a history of suspected or treated HCC.

## 2. Materials and Methods

Patients undergoing routine surveillance were prospectively recruited from October 2019 to October 2020 from a high-risk liver clinic within a single tertiary institution, Nepean Hospital, in Sydney, Australia. Inclusion criteria included age over 18 years and a current or suspected diagnosis of HCC or previously treated HCC. All patients provided informed consent and all patients were discussed as part of standard care by the multidisciplinary care team (MDT) comprised of radiologists, interventional radiologists, nuclear medicine physicians, gastroenterologists, radiation oncologists and upper GI surgeons. Exclusion criteria included patients unable to give informed consent and females of reproductive age.

The 68-Ga PSMA PET/CT scans were performed on a GE Discovery 64 PET/CT scanner with 68 Ga-labelled PSMA ligand N,N′-bis [2-hydroxy5-(carboxyethyl)benzyl] ethylenediamine-N,N′-diacetic acid, or HBED-CC [9]. Gallium-68 labelling was obtained using a Germanium-68/Gallium-68 radionucleotide generator and used for radiolabelling of PSMA-HBED-CC with an automated radio-synthesiser. Following intravenous tracer injection, dosed was determined according to patient weight: <60kg (200 MBq), 61–90 kg (250 MBq), >90 kg (300 MBq), and PET images were acquired 40 min after tracer injection over ~ 1 h. The images were fused with concurrent low dose CT images (120 keV and 60 mAs per section) for lesion localization and attenuation correction.

CT with arterial, portal-venous and delayed phase imaging was performed either immediately before or immediately after on the same scanner, as part of routine surveillance and reported separately to the PET/CT with the reader having access to the PET/CT images. The results of the previous or progress imaging including MRI within 3 months, tumour markers and clinical progress up to 12 months following recruitment were independently adjudicated according to concordance and compared with MDT assessments. The MDT assessment was considered a real-world standard of truth as histological diagnosis is not always obtained and often decision-making regarding HCC treatment is performed based on a composite of available clinical evidence [10,11]. The sensitivities, specificities, PPV and NPV were determined on a per-lesion basis. Each lesion was individually evaluated during follow-up. The progression of the patient as a whole was recorded as a better indicator of the accuracy of diagnosis and as progressive disease became more apparent with time. Lesions were still identified and individually assessed at MDT assessments and for the purpose of this exploratory trial. The study was approved by the Institutional Research Ethics Committee and all subjects provided informed consent to participate.

### Statistical Analysis

Data was analysed in Microsoft Excel (version 2012) for Windows 10. Cohorts were assessed for sensitivity, specificity, positive predictive value and negative predictive values compared to results from CT, MRI or histopathology. Where a comparison of discrete subsets was required, the students t-test with *p* value < 0.05 considered statistically significant. Pearson’s correlation coefficient was used to determine correlation between continuous variables.

## 3. Results

The baseline characteristics of 19 patients recruited for inclusion in the study are shown in Table 1. Most were male (95%) with mean age of 65 years. The aetiology of liver disease was predominantly hepatitis C and steatohepatitis while in a third of cases the cause was unknown. Additionally, 63% (12 patients) had known HCC under surveillance and 37% (7 patients) had a suspected new diagnosis of HCC. All patients had established early-stage cirrhosis (Child Pugh A/B).

The 49 lesions were assessed in 19 patients, of which 25 were treated previously. The results of sensitivity, specificity, positive predictive and negative predictive values are shown in Table 2. An independently adjudicated gold standard was determined of the diagnosis (suspicious for HCC/not suspicious for HCC) and based on the patient’s clinical progress and management to date, with the adjudicated diagnosis shown in Table 3. In particular, where discordant results were recorded between MRI and CT in 12 lesions, reference was made to multi-disciplinary team meeting documentation to determine the overall impression of a team of experts as the “final diagnosis”.

While all lesions were assessed by PSMA PET and diagnostic CT, MRI was only performed within 3 months of the PSMA PET/CT in 30/49 cases (61%). Of these lesions, MRI and PSMA PET were equivalent in performance with respect to sensitivity (91 vs. 87%), specificity (70 vs. 73%), positive predictive (71 vs. 76%) and negative predictive (90 vs. 85%) values. Details of treatment, size and imaging characteristics of each lesion are summarised in Table 3.

**Figure 1:**
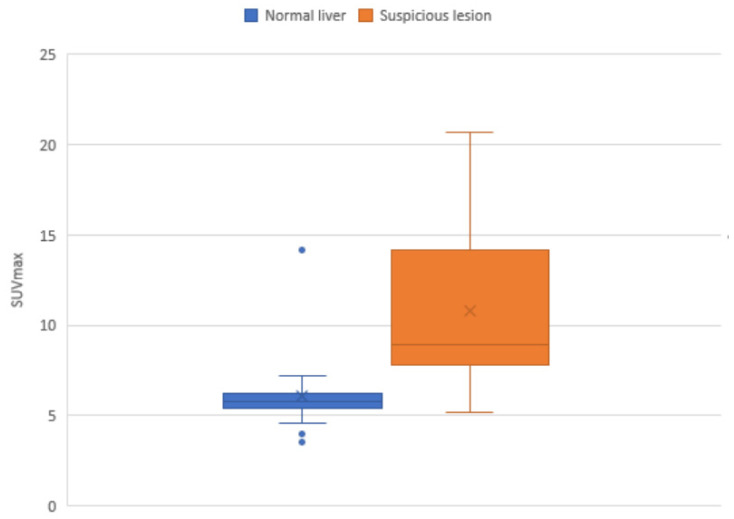
Interquartile SUVmax ranges for lesions considered suspicious based on PSMA, compared with average normal liver SUVmax. The blue dots are outliers.

**Figure 2:**
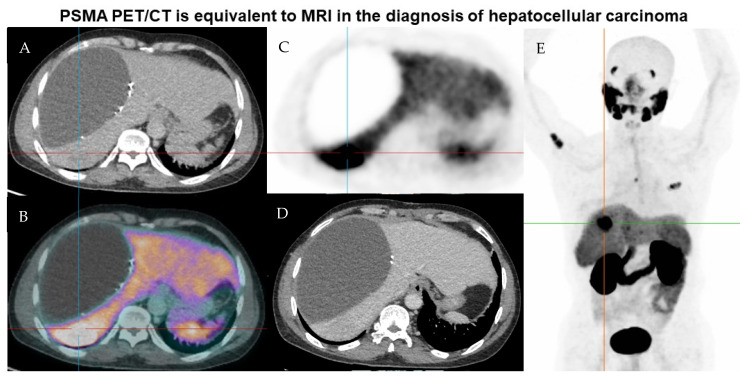
Representative imaging of a lesion suspicious for recurrence and metastatic spread of HCC on PSMA PET. Case details: 58-year-old male of Asian ethnicity with previous resection of HCC in 2019. (**A**) low dose CT, (**B**) fused PET PSMA and low does CT, (**C**) PSMA PET, (**D**) contrast-enhanced CT, (**E**) MIP PSMA PET image. There is a hypodense fluid attenuation area within the right liver adjacent to surgical clips representing a known seroma/biloma. Ultrasound guided biopsy of the PSMA avid lesion in hepatic segment 7 confirmed HCC. The patient was commenced on lenvatanib therapy with PSMA avid-likely bony metastatic disease in the right proximal humerus, T5 vertebral body and left 3rd rib anterolaterally. The liver lesion was non-enhancing on contrast-enhanced CT.

The imaging characteristics of PSMA uptake in benign and malignant liver conditions has previously been published by the research group using a large prospectively maintained database [12]. Figure 1 shows the relationship between normal liver and the SUVmax range of suspicious avid lesions on PSMA PET. Of the 49 lesions assessed, the average SUVmax in normal liver was 6.1 (SD 2.2, IQR 5.5–6.2) whereas in suspicious PSMA avid lesions, the average SUVmax was 10.8 (SD 4.9, IQR 8.0–11.6) with cross-over between groups. The difference in the mean SUVmax was statistically significant (*p* score = 0.0002). This may provide a threshold SUVmax for assessment of suspicious lesions. An example of a PSMA positive HCC is shown in Figure 2. 

In the cohort assessed, suspicious lesions as small as 5–6 mm were detectable on PSMA PET, although this is considered the lower limit of size for reliable resolution of lesions. There was a weak positive correlation between lesion size and SUVmax (Pearson r correlation coefficient 0.55). PSMA PET/CT also detected four lesions which were not found on MRI and CT, of which one of these was biopsy-proven to be HCC, leading to significant earlier management in 4/19 (21%) patients. Histological confirmation of lesions was pursued in a minority of cases 3/19 (16%) patients. A heterogeneous pattern of PSMA uptake was considered undetermined.

AFP was normal at the time of the PSMA scan in 13 out of 19 patients, with the actual AFP for each patient shown in Table 3, together with the peak AFP measured at any time point during the patient’s disease (including before inclusion in the study) shown in parenthesis.

## 4. Discussion

The utility of PSMA PET/CT for the diagnosis of pathologically confirmed HCC was previously described [13]. In this study by Jiao et al., PSMA uptake correlated with tumour vascularity in HCC. PSMA expression was seen on tumour vessels and on canalicular membrane of tumour cells [14] and PSMA expression appears to correlate positively with grade in HCC [15]. Other studies have also confirmed the usefulness of PSMA PET/CT in the detection of hepatocellular carcinoma and differentiating malignancy from changes of cirrhosis, with PSMA PET/CT considered at least as sensitive, if not superior to FDG PET/CT [8,14,15,16].

In the management of HCC, histopathological confirmation may not always be possible or clinically indicated due to the risk of seeding [4,5]. Imaging is therefore vital in the diagnosis of HCC, with the typical pattern of hyperenhancement in the arterial phase and washout in venous or delayed phases on contrast-enhanced CT or MRI [17,18]. In the current study, PSMA PET/CT had equivalent sensitivity and specificity to MRI (performed within 3 months of the PSMA PET/CT) in the detection of HCC and correlated well with clinical outcomes over a median period of follow-up of 6 months. In particular, where discordant results were recorded between MRI and CT in 12 lesions, the findings on PSMA PET/CT were in line with recommendations by the multidisciplinary care team and clinical management, suggesting possible utility for PSMA PET/CT as an additional imaging modality where equivocal results arise in high-risk patients, with lesions that may be amenable to percutaneous or surgical intervention. Due to waiting periods for imaging studies, 3 months was accepted as a time interval between PSMA PET and MRI for correlation to remain valid. This may be suboptimal given that progression in established HCC can occur rapidly, with the median survival in Australia for patients diagnosed with HCC being approximately 20 months [19]. However, this was likely a feasible time frame between initial imaging, MDT assessments and subsequent MRI imaging for complete assessment of lesions. 

False positives occurred in 8/49 lesions on PSMA PET/CT, higher than in the other imaging modalities, of which 6 occurred in previously treated lesions, possibly due to post-treatment inflammatory activity. In one patient, PSMA PET/CT detected undiagnosed skeletal metastases, for which localised radiotherapy and Lenvatinib therapy was prescribed, suggesting a potential role for PSMA PET/CT in the assessment for metastatic disease where conventional therapy would be unrevealing. Conversely, false negative results were lowest with PSMA PET/CT, occurring at a rate of 2/49 (4%) compared to MRI at a rate of 2/30 (7%), whilst CT had a false negative rate of 15/49 (31%).

The patient cohort consisted of 18 patients with previously treated suspected recurrent HCC and one patient with newly diagnosed primary HCC for work-up. The interval between most recent treatment for HCC and PSMA PET examination varied from 3.5 months to 32 months (average of 10.3 months). Given the predominance of patients being under surveillance for recurrent HCC, it is likely that the sensitivity and specificity demonstrated would be most applicable to this group.

PET imaging was previously utilised for the assessment of HCC. Other imaging agents such as FDG [20] and choline [21] have demonstrated complementary potential [22] in the detection of moderate to poorly differentiated HCC with a sensitivity of up to 75% in the detection of moderately differentiated HCC using choline [23]. Exploration using newer and accessible PET tracers may continue to improve our understanding of the role of molecular probes for HCC detection.

The strengths of the study include its integration in a real world setting and the accuracy of comparison with diagnostic CT performed concurrently with PSMA PET. A drawback of the study included that not all patients who performed PSMA PET also underwent MRI. Other weaknesses of the study include the lack of comparative ultrasound imaging, the relatively small sample size, predominance of patients being screened for recurrent HCC and the lack of a histological diagnosis in some cases.

Future studies are currently underway including Phase 2 studies [24] that will assess the utility of PSMA PET/MRI as a combined imaging modality [25] and may continue to shed more light on the utility of PSMA PET in the detection of HCC. PSMA-positivity may indeed provide options for theranostic approaches using molecular markers linked with radionuclide therapies [26].

## 5. Conclusions

In our exploratory study, PSMA PET/CT was just as sensitive as MRI in the detection of HCC and superior to CT. PSMA PET/CT may serve as a confirmatory test when results are equivocal from conventional imaging, allowing for earlier diagnosis and improved management for HCC.

## Figures and Tables

**Table 1:** Baseline characteristics.

Mean age (years)	65 (SD 7.2)
Male	18/19 (95%)
Average BMI (kg/m^2^)	31.5 (SD 6.7)
Aetiology of liver disease (/19 patients)	Hepatitis C (HCV): 5 (26%) Non-alcoholic steatohepatosis (NASH): 4 (21%) Alcoholic liver disease: 1 (5%) Mixed (HCV and NASH): 1 (5%) Mixed (HCV and alcoholic liver disease): 2 (11%) Unknown: 6 (32%)
Lesions	Total assessed: 49 Average lesion/patient: 2.6 *
Average PSMA activity administered (MBq)	285.7
Median AFP at time of study (normal reference ≤ 8 IU/mL)	5
Previously treated patients	12/19 (63%)
Median follow-up period	204 days

* In multi-focal disease, only the largest 5 lesions were assessed.

**Table 2:** Comparison of screening modalities.

(n)	PSMA (49)	MRI (30) *	CT (49)	Serum AFP (19) *	Average AFP Measurement (19) *
True negative	19	11	27		7
True positive	20	13	7		5
False negative	2	2	15		6
False positive	8	4	0		1
Sensitivity	91	87	32	45	
Specificity	70	73	100	88	
PPV	71	76	100	83	
NPV	90	85	64	54	

* MRI and serum AFP was not performed in every patient.

**Table 3:** Individual lesion assessment.

Lesion/Patient Initials	Size (Segment)	Previous Treatment	PSMA Uptake	MRI (Performed within 3 Months)	CT	AFP at Time of Scan (Peak) (RR ≤ 8)	Diagnosis (Obtained at MDT)	Progress (Months of Follow-Up Post Study)
1 BB	34 mm (7)	MWA	N	-	A+, PV+, D+	4 (30)	SD	SD (11.3)
2 BB	<5 mm (5)	N	N	-	A+, PV+, D+		SD	
3 GB	9 mm (7)	N	N	A+, DWI−	NS	6 (511)	SD	SD (0.5)
4 GB	9 mm (5/8)	MWA	N	A-, DWI−	NS		SD	
5 GB	19 mm (3)	MWA	N	A−, PV−	Hypodense		SD	
6 GB	<5 mm (7)	N	N	A+ (sus)	NS		SD	
7 HB	47 mm (8)	TACE	Heterogeneous	T2+, T1−, DWI+, A+, progressive washout (sus)	Hypodense, mild PV enhancement (sus)	197 (214)	PD	PD, palliative, deceased (12.5)
8 HB	17 mm (2)	N	Y (SUVmax 8.3)	T2+, progressive washout (sus)	NS		PD	
9 HB	11 mm (4A)	N	Y (SUVmax 8.9)	T2+, progressive washout (sus)	NS		PD	
10 HB	7 mm (6)	N	Y (SUVmax 10.1)	T2+, progressive washout (sus)	NS		PD	
11 HB	6 mm (8)	N	Heterogeneous	T2+, progressive washout (sus)	NS		PD	
12 JB	17 mm (7/8)	TACE	Y (SUVmax 5.2)	-	Isodense, areas of PV washout (sus)	23 (44)	PD	PD (9.6), retreated
13 KC	10 mm (4A/B)	Resected	Y (SUVmax 9.3)	-	NS	2 (3)	? PD	SD, false positive (6.4)
14 RC	38 mm (2)	MWA	N	-	Hypodense	7 (18)	SD	PD, false negative (6.9)
15 RC	30 mm (2/3)	TACE	Heterogeneous, SUVmax up to 14.2	-	Hypodense		SD	
16 DD	25 mm (4A)	TACE	Y (SUVmax 8.0)	T1−, T2+, A+, progressive washout (sus)	Hyperdense	326 (443)	? PD	PD (6.8)
17 DD	33 mm (7)	TACE	N	Heterogeneous, T1+, T2+, progressive washout, some peripheral enhancement (sus)	Hypodense		SD	
18 DD	37 mm (6)	-	Y (SUVmax 20.5)	T1−, T2+, DWI+, A+, progressive washout (sus)	NS		PD	
19 DD	12 mm (4B)	-	Y (SUVmax 5.9)	T1−, T2+, DWI+, A+, progressive washout (sus)	NS		PD	
20 GE	31 mm (5)	TACE	N	T1+, T2−, DWI−, A−	Hypodense, subtle arterial enhancement, no significant washout	1 (2)	SD	SD (3.5)
21 GE	Adjacent to lesion above (5/8)	-	Y (SUVmax 7.2)	T1+, T2−, DWI−, A−	Hypodense, subtle arterial enhancement, no significant washout		PD	
22 JF	20 mm (7)	TACE	N	No significant contrast enhancement	Hyperdense, A−	2 (7)	SD	PD (11.9)
23 JF	17 mm (1)	-	Y (SUVmax 8.1)	A+ (sus)	NS		PD	
24 JF	5 mm (5)	-	N	A+, rapid washout	NS		SD	
25 JF	22 mm (6)	MWA	N	A+, rapid washout	A+, no significant washout		SD	
26 JF	5 mm (7)	-	N	A+, rapid washout	NS		SD	
27 RH	25 mm (7)	SBRT	Y (SUVmax 12.7)	-	Hypodense	35 (1196)	SD	SD, false positive (2.3)
28 NK	37 mm (3)	MWA	Y (SUVmax 11.0)	-	A−, no significant portal venous enhancement	3 (3)	SD	SD, false positive (6.0)
29 NK	10 mm (7)	-	Y (SUVmax 10.7)	-	NS		SD	
30 RL	50 mm (8)	MWA	N	Areas of peripheral T1+ with central T1 isointensity, central T2+ and peripheral T2−, subtle restricted diffusion (sus)	Low attenuation	2 (4)	SD	SD (6.3)
31 RM	23 mm (6)	TACE	Y (SUVmax 6.4)	No enhancement	No significant enhancement	3 (220)	SD	SD, false positive (12.5)
32 RM	8.4 mm (6/7)	-	N	A+, washout with normalisation	No significant enhancement		SD	
33 JM	18 mm (8)	MWA	N	-	No significant enhancement	15 (15)	SD	PD, false negative (4.5)
34 JM	13 mm (6)	TACE	N	-	NS		PD	
35 JM	12 mm (2)	-	N	-	NS		SD	
36 JM	5 mm (4A)	-	N	-	NS		SD	
37 JM	5 mm (4B)	-	N	-	NS		PD	
38 JM	5 mm (4A/8)	MWA	Y (SUVmax 8.4)	Delayed hypointensity	NS	5 (7)	PD	PD (9.9)
39 JM	32 mm (7)	MWA	N	Small T1+ hyperintensity, A-	NS		SD	
40 FP	33 mm (8)	MWA	Y (SUVmax 11.6)	A−, hypointense on PV and hepatocyte phase imaging	No significant enhancement	3 (4)	PD	PD (7.0)
41 RS	19 mm (2)	-	Y (SUVmax 10.0)	Mildly T2−, isointense T1 FS, DWI+, peripheral A+ and early washout (sus)	Enlarging, low attenuation with A+ and washout (sus)	18 (30)	PD	PD (8.1)
42 RS	38 mm (5)	-	Y (SUVmax 16.7)	Mildly T2-, isointense T1 FS, DWI+, peripheral A+ and early washout (sus)	Low attenuation with A+ and washout (sus)		PD	
43 RS	19 mm (6)	-	Y (SUVmax 9.6)	Mildly T2−, isointense T1 FS, DWI+, peripheral A+ and early washout (sus)	Low attenuation with A+ and washout (sus)		PD	
44 RS	27 mm (5/8)	-	Y (SUVmax 17.4)	Mildly T2−, isointense T1 FS, DWI+, peripheral A+ and early washout (sus)	Enlarging, low attenuation with A+ and washout (sus)		PD	
45 KU	10 mm (6)	-	Y (SUVmax 7.6)	-	Subtle arterial enhancement with washout on delayed phase (sus)	2 (32)	PD	PD (3.6)
46 KU	19 mm (6)	MWA	Y (SUVmax 8.1)	-	Hypointense, non-enhancing		SD	
47 KU	20 mm (4A)	-	Y (SUVmax 7.5)	-	Hypointense, non-enhancing		SD	
48 KU	34 mm (5/8)	MWA	Y (SUVmax 7.4)	-	Hypointense, non-enhancing		SD	
49 SW	29 mm (7)	MWA	Y (SUVmax 20.7)	-	Non-enhancing	1 (2)	PD	PD (5.7)

MWA: microwave ablation, TACE: trans-arterial chemoembolization, SBRT: single beam radiotherapy; A+: arterial enhancement; A−: no arterial enhancement; T1/2+: hyperintense; T1/2−: hypointense; PV: portal-venous; PD: progressive disease; SD: stable disease; Y: positive for PSMA uptake; N: negative for PSMA uptake; SUVmax: maximum standardized uptake value.

## Data Availability

All data analysed is shown in Table 3.
